# Rheumatology training experience across Europe: analysis of core competences

**DOI:** 10.1186/s13075-016-1114-y

**Published:** 2016-09-23

**Authors:** Francisca Sivera, Sofia Ramiro, Nada Cikes, Maurizio Cutolo, Maxime Dougados, Laure Gossec, Tore K. Kvien, Ingrid E. Lundberg, Peter Mandl, Arumugam Moorthy, Sonia Panchal, José A. P. da Silva, Johannes W. Bijlsma, the Working Group on Training in Rheumatology across Europe, Ledio Ҫollaku, Armine Aroyan, Helga Radner, Anastasyia Tushina, Ellen De Langhe, Sekib Sokolovic, Russka Shumnalieva, Marko Baresic, Ladislav Senolt, Mette Holland-Fischer, Mart Kull, Antti Puolitaival, Nino Gobejishvili, Axel Hueber, Antonis Fanouriakis, Paul MacMullan, Doron Rimar, Serena Bugatti, Julija Zepa, Jeanine Menassa, Diana Karpec, Snezana Misevska-Percinkova, Karen Cassar, Elena Deseatnicova, Sander W. Tas, Elisabeth Lie, Jan Sznajd, Florian Berghea, Anton Povzun, Ivica Jeremic, Vanda Mlynarikova, Mojca Frank-Bertoncelj, Katerina Chatzidionysiou, Alexandre Dumusc, Gulen Hatemi, Erhan Ozdemirel, Iuliia Biliavska

**Affiliations:** 1 Department Reumatologia, Hospital General Universitario de Elda, ctra Sax s/n, Elda, Alicante, 03600 Spain; 2 Leiden University Medical Center, Leiden, The Netherlands; 3 University of Zagreb School of Medicine, University Hospital Centre, Zagreb, Croatia; 4 Research Laboratories and Academic Division of Clinical Rheumatology, Postgraduate School on Rheumatology, Department of Internal Medicine University of Genova, Genova, Italy; 5 Université Paris Descartes University, Department of Rheumatology, Hôpital Cochin, Assistance Publique-Hôpitaux de Paris; INSERM (U1153): Epidemiologie Clinique et Biostatistiques, PRES Sorbonne Paris-Cité, Paris, France; 6 Sorbonne Universités, UPMC Univ Paris 06, Institut Pierre Louis d’Epidémiologie et de Santé Publique; AP-HP, Pitié Salpêtrière Hospital, Department of rheumatology, F-75013 Paris, France; 7 Department of Rheumatology, Diakonhjemmet Hospital, Oslo, Norway; 8 Rheumatology Unit, Department of Medicine, Karolinska University Hospital, Solna, Sweden; 9 Karolinska Institutet, Stockholm, Sweden; 10 University of Vienna, Vienna, Austria; 11 University Hospitals of Leicester, Leicester, UK; 12 University Hospitals of Leicester NHS Trust, Leicester, UK; 13 Centro Hospitalar e Universitário de Coimbra, Faculdade de Medicina da Universidade de Coimbra, Coimbra, Portugal; 14 University Medical Center Utrecht, Utrecht, The Netherlands

**Keywords:** Training, Rheumatology, Europe, UEMS, Competence, Education, Assessment

## Abstract

**Background:**

The aim of this project was to analyze and compare the educational experience in rheumatology specialty training programs across European countries, with a focus on self-reported ability.

**Method:**

An electronic survey was designed to assess the training experience in terms of self-reported ability, existence of formal education, number of patients managed and assessments performed during rheumatology training in 21 core competences including managing specific diseases, generic competences and procedures. The target population consisted of rheumatology trainees and recently certified rheumatologists across Europe. The relationship between the country of training and the self-reported ability or training methods for each competence was analyzed through linear or logistic regression, as appropriate.

**Results:**

In total 1079 questionnaires from 41 countries were gathered. Self-reported ability was high for most competences, range 7.5–9.4 (0–10 scale) for clinical competences, 5.8–9.0 for technical procedures and 7.8–8.9 for generic competences. Competences with lower self-reported ability included managing patients with vasculitis, identifying crystals and performing an ultrasound. Between 53 and 91 % of the trainees received formal education and between 7 and 61 % of the trainees reported limited practical experience (managing ≤10 patients) in each competence. Evaluation of each competence was reported by 29–60 % of the respondents. In adjusted multivariable analysis, the country of training was associated with significant differences in self-reported ability for all individual competences.

**Conclusion:**

Even though self-reported ability is generally high, there are significant differences amongst European countries, including differences in the learning structure and assessment of competences. This suggests that educational outcomes may also differ. Efforts to promote European harmonization in rheumatology training should be encouraged and supported.

**Electronic supplementary material:**

The online version of this article (doi:10.1186/s13075-016-1114-y) contains supplementary material, which is available to authorized users.

## Background

Rheumatology specialty training is the educational process required for a physician to be officially recognized as a specialist in rheumatology. In each country, it is defined by one (or sometimes several) officially approved training programs, which aim to bring physicians to an agreed standard of proficiency in the management of patients with rheumatic and musculoskeletal diseases (RMDs). The development and implementation of these training programs are performed under the national authorities. Data on the scope of diseases managed by rheumatologists in each country are lacking, but the national training programs might well reflect the differences in the local definition of a rheumatologist.

Harmonization of specialty training has been considered essential not only to support the free movement of rheumatology specialists within Europe, but also to facilitate equal standards of care for patients with RMDs. Multinational recommendations on rheumatology training are scarce. In the European Union a minimum duration of four years for rheumatology training programs [1] has been established and the Rheumatology Board and Section of the European Union of Medical Specialists (UEMS) has developed recommendations on the structure and content of the training programs [2–4]. The implementation of these recent recommendations, however, remains at the discretion of the individual countries. There has been very little analysis of the current training situation [5, 6].

In a previous study, we assessed the training regulations throughout Europe with regards to length, structure of training, contents and required assessments [7]. Forty-one European countries offer rheumatology training programs, usually organized at a national level, but with substantial variations in all aspects analyzed [7]. Though this prior study provides insight into the training regulations, training programs can be implemented in a variety of ways, as the governing principles are open to interpretation. The delivery of the same (national) training program can therefore vary widely between training centers. There are few comparative data on educational outcomes (i.e., in the competences actually acquired) [8].

The aim of this project, supported by the European League Against Rheumatism (EULAR), was to analyze and compare the educational experience in rheumatology specialty training programs across European countries, specifically looking at competences achieved, and the methods by which they are acquired (education and patient experience) and assessed.

## Methods

The current study was a cross-sectional survey. A Steering Group (SG) comprising 12 European rheumatologists with an interest in education agreed upon the main aspects of training to be assessed, after analysis of national training programs across Europe [7] and of the UEMS European Rheumatology Curriculum Framework [3]. A representative from each of the relevant EULAR member countries (national principal investigator (PI)) was identified and oversaw the dissemination of the survey among the target population of the PI’s country. These PIs constituted the Working Group. Given that the study consisted of an online survey among young rheumatologists and researchers, and no patients were involved, no ethics committee approval was required.

### Target population

The survey targeted rheumatologists who had been certified in the past five years and rheumatology trainees who were already in the rheumatology-specific part of their training in any of the 41 EULAR countries offering rheumatology specialty training. Rheumatology trainees who were still in their internal medicine training were excluded. The estimated target population included approximately 4500 young rheumatologists [7]. This estimation was obtained by multiplying the number of trainees who started the training in a given country in the year before the survey (as determined by the national PI) times the number of years of the rheumatology-specific period of training plus five years (in order to include rheumatologists certified in the past five years).

### Survey content

Through an iterative voting process, the SG selected 21 core competences, which formed the basis of the survey and could be divided into three main areas: (1) Clinical competences: both broad clinical competences ((a) performing a clinical examination; (b) detecting synovitis of metacarpophalangeal and proximal interphalangeal joints; (c) interpreting specific laboratory tests; and (d) applying common measures of disease activity) and disease-specific clinical competences ((a) performing the initial diagnostic and management approach to a patient with a single swollen joint; and managing a patient with (b) osteoarthritis (OA); (c) gout; (d) early rheumatoid arthritis (RA)/undifferentiated arthritis; (e) spondyloarthritis (SpA); (f) autoimmune connective tissue diseases (CTD); (g) systemic vasculitis; (h) osteoporosis (OP); and (i) initiating and monitoring therapy with a biologic agent); (2) Procedures: (a) performing knee aspiration; (b) identifying crystals on an optic microscope; (c) interpreting a conventional hand x-ray; (d) performing musculoskeletal (MSK) ultrasound (US); and (3) Generic competences: (a) working within a multidisciplinary team; (b) interpreting the results of a scientific paper; (c) giving a scientific presentation; and (d) communicating with patients and families).

On each competence, information was gathered on self-reported ability through an 11-point numerical rating scale (NRS) where 0 meant “unable to do” and 10 meant “fully able to do independently”. Respondents were considered to have low self-reported ability if the NRS score was <5 and very low self-reported ability if the NRS score was <3. Information was also gathered on the training process for each competence (where applicable), specifically on the existence of formal education (yes/no) and on the number of patients (personally) managed during training in the pre-set categories (0, 1–10, 11–50, 51–100, 101–150, or >150 patients). Respondents were considered to have limited patient experience if they had managed ≤10 patients/procedures. Finally, data were gathered on whether each competence was formally assessed. Additional questions on research and on the existence of a log-book were included. The complete survey can be found in Additional file 1.

All questions referred to the end of the training period. Trainees were asked to answer based on the reasonable expectations of their training. Qualified rheumatologists were asked to reflect their status at the end of their training period. Data collection took place from June to December 2014.

### Data analysis

Descriptive statistics were used to summarize the data. Self-reported ability on each of the competences was compared between groups: respondents who received formal education vs. those who did not, respondents with limited patient experience vs. those with wider experience, respondents assessed vs. those not assessed, respondents from countries with short training vs. long training, and respondents from countries with explicit inclusion of competence in the training program vs. those without inclusion. These comparisons were performed using the Mann-Whitney *U* test (variables non-normally distributed). In order to investigate whether the country of training was associated with differences in self-reported ability, training methods (education, patient experience) or assessment frequency for each competence we conducted multivariable regression analysis (linear or logistic, for continuous or categorical outcomes, respectively). Four models were therefore constructed for each competence, with country of training as the main variable of interest. Analyses were adjusted for the respondent’s gender, age, certification status, and explicit inclusion of the respective competence in the country’s official training program. The model for self-reported ability was also adjusted for the existence of formal education, for limited practical experience, and for assessment; the model for assessment was adjusted for formal education and for limited patient experience. For self-reported ability, alternative models with total length of training instead of country were computed and the model fit was compared using the *R*^2^ statistic. In the multivariable models, the UK was chosen as the reference country. Data on training length and inclusion of the competence in the curriculum was obtained from prior work by our group [7]. Analyses were repeated including only UEMS member countries. All analyses were performed with Stata SE V.12 (StataCorp, College Station, TX, USA).

## Results

In total, 1433 responses were received. After deleting incomplete responses (only demographic data provided) and responses outside the target population (e.g., trainees prior to the rheumatology-specific period of the training or rheumatologists qualified over five years ago), 1,079 responses were included in the final analyses. Respondents were mostly women and were evenly distributed between trainees and certified rheumatologists (Table 1).

**Table 1:** Demographic characteristics of the 1079 survey participants

Characteristic		Value, n(%)
Gender	Male	323 (30 %)
	Female	753 (70 %)
Age	≤25 years	20 (2 %)
	26–30 years	260 (24 %)
	31–35 years	389 (36 %)
	36–40 years	274 (25 %)
	>40 years	136 (13 %)
Trainees (year of training)	Total	559 (52 %)
	1st year	54 (5 %)
	2nd year	78 (7 %)
	3rd year	159 (15 %)
	4th year	129 (12 %)
	5th year	65 (6 %)
	6th year	51 (5 %)
	7th year	13 (1 %)
	No information available	10 (1 %)
Rheumatologist (years since qualification)	Total	520 (48 %)
	Year 1	160 (15 %)
	Year 2	104 (10 %)
	Year 3	102 (9 %)
	Year 4	75 (7 %)
	Year 5	58 (6 %)
	No information available	21 (2 %)

The overall response rate was 24 % of the estimated target population. All 41 countries provided responses, but the response rate varied widely amongst countries from 3 % (Belarus) to 100 % (Bosnia & Herzegovina and Estonia). Thirty-six countries achieved a response rate >10 % (all except Armenia, Belarus, Germany, Italy and Ukraine). See Additional file 2 for more details.

### Self-reported ability

Mean self-reported ability was high at the end of the training period in most of the 13 clinical competences, ranging from 7.5 (managing a patient with vasculitis) to 9.4 (interpreting laboratory tests) (Table 2). Self-reported ability in the different procedures and techniques was variable, being high for knee aspiration (9.0) and interpreting hand x-rays (8.2) but low for crystal identification (6.1) and performing musculoskeletal (MSK) US (5.8). In general, self-reported ability in generic competences (range 7.8–8.9) was similar to ability in clinical competences.

**Table 2:** Description of self-reported ability, formal education, patient experience and assessment of 21 core competences

	Self-reported ability (score 0–10) (mean (SD))	Low self-reported ability (NRS score <5)	Formal education^b^ (yes)	Limited patient experience^c^ (≤10)	Assessment^d^ (yes)
Musculoskeletal exam	9.0 (1.5)	18 (1.7 %)	785 (75 %)	NR	517 (51 %)
Detection of synovitis	9.0 (1.4)	20 (1.9 %)	783 (75 %)	NR	521 (52 %)
Mono-arthritis	9.1 (1.4)	15 (1.4 %)	831 (80 %)	126 (12 %)	569 (57 %)
Laboratory test interpretation	9.4 (1.1)	8 (0.7 %)	836 (81 %)	NR	578 (58 %)
Osteoarthritis^a^	8.9 (1.6)	25 (2.3 %)	764 (74 %)	70 (7 %)	519 (52 %)
Gout^a^	9.1 (1.4)	18 (1.7 %)	855 (83 %)	144 (14 %)	544 (54 %)
Early rheumatoid arthritis^a^	9.0 (1.4)	18 (1.7 %)	925 (89 %)	97 (10 %)	604 (60 %)
Spondyloarthritis^a^	9.0 (1.4)	13 (1.2 %)	939 (91 %)	83 (8 %)	597 (60 %)
Autoimmune connective tissue diseases^a^	8.0 (1.9)	60 (5.6 %)	821 (79 %)	214 (21 %)	568 (57 %)
Vasculitis^a^	7.5 (2.2)	101 (9.4 %)	768 (74 %)	450 (44 %)	513 (51 %)
Osteoporosis^a^	8.7 (1.6)	29 (2.7 %)	816 (79 %)	93 (9 %)	536 (53 %)
bDMARD^a^	8.8 (1.8)	43 (4.0 %)	814 (78 %)	170 (17 %)	527 (52 %)
Use of disease activity measures	8.9 (1.5)	22 (2.1 %)	802 (77 %)	NR	537 (54 %)
Knee aspiration	9.0 (2.0)	59 (5.5 %)	828 (80 %)	186 (18 %)	483 (48 %)
Crystal identification	6.1 (3.8)	346 (32.3 %)	545 (53 %)	622 (61 %)	295 (29 %)
X-ray interpretation	8.2 (1.9)	54 (5.1 %)	725 (70 %)	104 (10 %)	473 (47 %)
Ultrasound	5.8 (3.4)	342 (32.2 %)	721 (70 %)	391 (39 %)	434 (43 %)
Multidisciplinary team	8.1 (2.3)	86 (8.1 %)	NR	NR	376 (37 %)
Interpret published paper	7.8 (2.1)	85 (7.9 %)	648 (62 %)	NR	407 (41 %)
Presentation	8.1 (2.1)	83 (7.8 %)	618 (59 %)	408 (40 %)	472 (47 %)
Communication	8.9 (1.5)	23 (2.1 %)	547 (53 %)	NR	418 (42 %)

Results presented as n (%) unless otherwise specified. ^a^Theses competences refer to managing a patient with the given disease/therapy. ^b^Formal education refers to respondents who had received any formal education such as courses, lectures, etc. ^c^Patient experience refers to number of patients (personally) managed during training. ^d^Assessment refers to respondent being assessed in the given competence. *bDMARD* biologic disease-modifying anti-rheumatic drug, *NRS* numerical rating scale, *NR* not reported

A proportion of trainees reported low confidence (NRS score <5) or even very low confidence (NRS score <3) in their ability in all core rheumatologic competences (Table 2 and Additional file 3). The percentage of respondents with low self-reported ability (NRS score <5) ranged in clinical competences from 1 % (interpreting laboratory tests) to 9 % (managing a patient with vasculitis), in procedures from 5 % (interpreting hand x-rays) to 32 % (identifying crystals under a microscope) and in generic competences from 2 % (patient communication) to 8 % (participating in a multidisciplinary team).

### Training process (education and patient experience)

More than 70 % of the respondents felt that they had received formal education in each of the clinical competences (Table 2). In contrast, the only procedure in which >70 % of respondents had received formal education was knee aspiration (80 %). The proportion of respondents reporting formal education in generic competences was also lower than in clinical competences (53–62 %).

A proportion of respondents managed ≤10 patients with each disease analyzed, ranging from 7 % of respondents managing ≤10 patients with OA to 44 % managing ≤10 patients with vasculitis. The proportion of respondents who performed ≤10 procedures during their training was smaller for knee aspiration (18 %), and hand x-ray interpretation than for US (39 %) and crystal identification (61 %). Forty percent of trainees performed ≤10 scientific presentations during their training.

A total of 844 (83 %) respondents were running a quasi-independent outpatient clinic by the end of their training. Similarly, 835 (82 %) of the respondents initiated and/or monitored patients under treatment with bDMARDs and 936 (92 %) reported using disease activity measures regularly. Half (n = 499) of the respondents published a research paper as a first or second author during their training.

### Assessment of competences

Around half the respondents (51–60 %) reported undergoing an assessment in each of the clinical competences (Table 2). A smaller proportion of respondents were assessed in practical procedures and generic competences (29–48 %), with the smallest percentage of assessments being performed for crystal identification. Sixty percent (n = 595) of survey respondents reported keeping a log-book or portfolio in which training activities were registered.

### Self-reported ability in different subgroups

Mean self-reported ability in all 21 core competences was higher in the group of respondents who received formal education than in those who did not (Table 3). In a similar manner, the mean self-reported ability in the group of respondents who had limited practical experience (who had managed ≤10 patients) was lower than in those who had greater practical experience (Additional file 4). However, respondents who were assessed only had higher mean self-reported ability in 12 of the competences assessed: performing a MSK examination, using disease activity measures, managing patients with OA, gout, CTD, vasculitis, and OP, identifying crystals, interpreting a hand x-ray, performing US exam, interpreting a research paper and performing a scientific presentation. In the remaining eight competences, the mean self-reported ability in respondents who had been assessed was not different to the mean in those who had not been assessed (Additional file 5).

**Table 3:** Comparison of self-reported ability in each competence in respondents receiving formal education and those not receiving formal education

	Self-reported ability in respondents without formal education (mean (SD))	Self-reported ability in respondents receiving formal education (mean (SD))	*P* value
Musculoskeletal exam	8.5 (1.7)	9.1 (1.4)	<0.0001
Detection of synovitis	8.8 (1.5)	9.1 (1.4)	0.0052
Mono-arthritis	8.8 (1.5)	9.2 (1.3)	0.0002
Laboratory test interpretation	9.2 (1.2)	9.5 (1.0)	0.0001
Osteoarthritis^a^	8.6 (1.7)	9.0 (1.5)	<0.0001
Gout^a^	8.9 (1.5)	9.2 (1.3)	<0.0001
Early rheumatoid arthritis^a^	8.8 (1.58)	9.1 (1.3)	<0.0001
Spondyloarthritis^a^	8.7 (1.6)	9.1 (1.3)	0.0049
Autoimmune connective tissue diseases^a^	7.7 (2.1)	8.1 (1.8)	<0.0001
Vasculitis^a^	7.0 (2.3)	7.6 (2.1)	<0.0001
Osteoporosis^a^	8.3 (1.8)	8.8 (1.5)	<0.0001
bDMARD^a^	8.5 (2.2)	9.0 (1.6)	0.0005
Disease activity measures	8.6 (1.8)	9.0 (1.4)	0.0004
Knee aspiration	8.6 (2.4)	9.1 (1.9)	<0.0001
Crystal identification	5.7 (3.9)	6.1 (3.8)	<0.0001
X-ray	7.8 (2.2)	8.3 (1.8)	<0.0001
Ultrasound	5.3 (3.5)	6.0 (3.4)	<0.0001
Multidisciplinary team	NR	NR	
Interpret published paper	7.5 (2.2)	8.0 (2.0)	<0.0001
Presentation	7.6 (2.4)	8.2 (2.0)	<0.0001
Communication	8.8 (1.5)	9.0 (1.5)	0.0080

^a^These competences refer to the management of a patient with the given disease or treatment. *bDMARD* biologic disease-modifying anti-rheumatic drug *NR* not reported

Longer training was associated with higher mean self-reported ability in all competences except for managing patients with vasculitis (Additional file 6), while the explicit inclusion of the individual competences in the national training program was associated with higher self-reported ability in only some procedures and generic competences, but not in clinical competences (Additional file 7).

### Association between country of training and self-reported ability and training methods

Through regression analysis, we identified factors associated with self-reported ability for each competence (separate models). Individual country data are available in Additional file 8. In an adjusted model (overall *p* value for the dummy variables of country) the country of training was significantly associated with self-reported ability in all 21 core competences (Additional file 9). For example, trainees from the UK reported (on average) 2.6 points higher ability in the management of patients with early RA than a trainee from Albania and 1.4 points higher than a trainee from France. In all 21 competences, formal education and managing >10 patients during training were also associated with increased self-reported ability. On the other hand, the undergoing an assessment was associated with increased self-reported ability in only six of the 21 competences analyzed (using disease activity measures, managing a patient with OA, CTD, or vasculitis, performing US exam and participating in a multidisciplinary team). The length of training was also associated with increased self-reported ability in all competences, but the models had a worse fit compared to the models with country.

In separate and adjusted regression models, the country of training was associated with differences in the odds of receiving formal theoretical education, of having limited patient experience and of undergoing an assessment in any given competence (Additional file 9). All analyses were repeated including only countries that are members of UEMS, and the results were consistent.

## Discussion

In this study we have explored the training experience of young European rheumatologists and trainees from the 41 countries where training is offered. Self-reported ability appears moderately high for most core competences, though no external reference has been found for comparison, not even in other medical specialties. Still, there is room for improvement. Moreover, differences between countries persist even after adjustment. If this reflects real differences in competence achieved during specialty training, the trans-national standard of care for patients with RMDs might be compromised.

Several educational “red flags” have been detected. There was consistently poor performance in the identification of crystals by optic microscopy: self-reported ability was low, as was the percentage of trainees receiving education and “sufficient” practical experience. Crystal identification is a mandatory technique for rheumatology trainees according to the UEMS recommendations [3] and to most national training programs. Whether the lack of competence is due to other specialists/laboratory technicians taking over the procedure is unknown; this approach, however, has low reliability [9, 10]. A second procedure - performing MSK US – was also poorly performed, with low self-reported ability in this procedure. This might reflect the prolonged learning curve for US, as those completing training might not yet have attained sufficient proficiency. However, US is not (yet) considered a mandatory competence at a European level or in many national curricula so the low self-reported ability could result from lack of access [11] or the acquisition of the competence by only some of the trainees. Surprisingly, ability even in what could be considered a core rheumatologic procedure - knee aspiration – was not optimal, with over 5 % of responders reporting low ability. Not being able to perform knee aspiration adequately could have a negative impact on other competences such as management of a patient with different types of arthritis or identifying crystals under the microscope. If global deficiencies are to improve, analysis of the causes by national and international organizations should be prioritized and solutions such as further promotion of courses or educational stays at reference centers considered.

Self-reported ability was better for clinical competences than for generic competences. The lowest self-reported ability within the disease areas was in managing patients with CTD, especially those with systemic vasculitis. This could be due to several reasons as these are fewer common diseases with potential multi-organ involvement, which can therefore present in a variety of manners. Also, rheumatologists might not be the main providers of care for these patients in some countries, adding to the European diversity. This issue was not addressed in this study.

Beyond global deficiencies, country-specific strengths and weaknesses have also been detected. In order to interpret the results a minimum background knowledge of rheumatology training programs across Europe is needed. In all countries, a period of general internal medicine training is required, though the timing of the training in internal medicine varies (within or prior to the rheumatology training program). Training length (considered as any prior internal medicine training and the rheumatology training program) will span from 3 to over 8 years. Given the differences in structure, this period provides a better reflection of true training time than the length of the rheumatology training program.

We accepted a broad definition of rheumatology for this project, but diversity in clinical rheumatology practice was documented over two decades ago [12]. This will impact the clinical competences and procedures that each country classes as mandatory during the training. The competences were chosen deliberately to cover a broad spectrum of rheumatology, but in order to maximize participation, the number of competences analyzed was necessarily limited.

Apart from the differences in self-reported ability, some countries have greater reliance on personal study (limited formal education), while in others the trainees are expected to see and manage a greater number of patients. As expected, greater experience increased the self-reported ability of trainees, as did receiving formal education. Even though didactic lectures do not modify physician behavior in continuous medical education, it must be noted that formal education can take different forms, including interactive formats, which do have behavior-modifying potential [13]. Also, postgraduate training comprises a critical educational time, with a need to incorporate vast amounts of knowledge in a relatively short time. The effect of assessments on self-reported ability was inconsistent. However, the type of assessment was not recorded and therefore anything from case discussions to written multiple-choice exams or objective structured clinical examination could be considered an assessment.

Even though comprehensive discussions were performed during the development and dissemination of this survey, several limitations must be noted. First, in this study we used self-reported ability - also referred to as confidence - as a surrogate marker for competence. In the absence of a pan-European examination, it is impossible to obtain a homogeneous assessment of competence, and self-reported ability could be a reasonable surrogate. However, it should be noted that this is a subjective assessment made by the young rheumatologist/trainee and thereby influenced by many external factors such as the referent against which it is compared (i.e., the standard the respondent considers optimal), the social context (e.g., the attitudes towards medical education), and personal traits (e.g., self-esteem).

Second, we asked trainees to estimate outcomes for the end of their training and rheumatologists to recall the end of their training. This introduces several biases (such as recall bias for young rheumatologists) and imprecision. Also, the survey captured answers from around a quarter of the target population; it is plausible that trainees who answered the survey are more engaged and optimistic than the general population. The limited number of participants, particularly from some countries, poses some challenges in the interpretation of the results. Country of training consistently and significantly played a role and analyses are robust for this conclusion. Nevertheless, due to a limited number of participants per country and particularly in some countries, the coefficients reflecting the magnitude of the difference between countries may be unstable, and therefore must be interpreted with caution.

Finally, some of the questions could have led to differences in interpretation. For this, we piloted the survey and provided definitions and examples of all terms considered less clear. For example we provided examples of what should be considered as formal education: “This does not mean that you have seen, managed or discussed patients with these complaints or that you can treat patients with these diseases. Rather we are asking if you have received formal education such as courses, lectures, etc”.

This study suggests that there are significant differences between European countries, not only in training structures and methods, but also in educational outcomes. The differences between countries can be attributed to a number of factors. Length of training does not fully explain the differences between countries. Other factors not assessed in this study (e.g., type of supervision, health care organization, etc.) are potential confounders of this relationship with length of training and should be explored to provide further insight into the differences between countries and the possible intervention strategies.

Homogeneous training might be considered unnecessary or even unwarranted, as national needs differ. However, harmonized training has been encouraged by all European authorities as an essential way to support doctor mobility within the European Union, improve national training, and assure trans-national standards of care for patients. Efforts towards this end have been ongoing for decades in many specialties [14–19]. In rheumatology, the Section and Board of the UEMS has produced several Charters and Educational Training Requirements, and the uptake of these, though currently limited, should be supported.

Other specialties have devised different paths to enhance harmonization, with greater or lesser success. Up to 30 UEMS specialty Boards are providing a (voluntary) European assessment [20]. Even though these are not to be considered as formal specialist qualifications, their quality and recognition have increased significantly and as a result, some countries recognize European assessments as part of (or equivalent to) their national examination. Furthermore, their mere existence has led to an increase in the number of trainees and specialists taking these exams, contributing to a higher standard of knowledge and competence, which may ultimately contribute to better delivery of care. Steps towards an assessment tool that can be used across all the USA rheumatology training programs have also been recently forthcoming [19]. Within the rheumatology community, further steps in this direction are needed; this can only take place with the involvement and commitment of the different countries and stakeholders.

In summary, this study reports that even though overall high self-reported ability is reported for most competences, significant differences in outcome and training methods remain amongst countries. Given the relevance of the issue, further analyses of the extent of the differences in achieved competences are warranted. Initiatives such as the development and implementation of a consensus list of core competences or a multinational examination should be supported and encouraged. Increased knowledge about national training provides the background information necessary to plan successful harmonization attempts.

## Conclusions

Trainees across Europe achieve high self-reported ability in most rheumatology competences, but several “red flags” have been identified. Self-reported ability of trainees and training methods differ among European countries. Further harmonization of competences across Europe should be supported to optimize the standard of care.

## Additional files

Additional file 1: Survey of the project “Assessment of training for Rheumatology fellows across Europe”. (DOCX 28 kb) Additional file 2: Table S1. Response numbers and rate per country. (DOCX 16 kb) Additional file 3: Table S2. Number and percentage of respondents with very low self-reported ability per competence. (DOCX 14 kb) Additional file 4: Table S3. Comparison of self-reported ability in each competence in respondents managing ≤10 patients and those managing >10 patients in the corresponding competence during their training. (DOCX 15 kb) Additional file 5: Table S4. Comparison of self-reported ability in each competence in respondents with and without assessment in the corresponding competence. (DOCX 15 kb) Additional file 6: Table S5. Comparison of self-reported ability in each competence in respondents with a long and short length of training (previous training in internal medicine plus rheumatology training program). (DOCX 15 kb) Additional file 7: Table S6. Comparison of self-reported ability in each competence in respondents whose national training program explicitly included the competence and those whose national training program did not. (DOCX 15 kb) Additional file 8: Table S7. Self-reported ability per individual country. (DOCX 23 kb) Additional file 9: Table S8. Factors associated with self-reported ability and with limited experience in early rheumatoid arthritis/undifferentiated arthritis (≤10 patients) and in performing knee aspiration (≤10 procedures). (DOCX 18 kb)

## Abbreviations

bDMARDs: biologic disease-modifying anti-rheumatic drugs
CoBaTrICE: competence-based training in intensive care medicine
CTD: autoimmune connective tissue diseases
EULAR: European League Against Rheumatism
MSK: musculoskeletal
NRS: numerical rating scale
OA: osteoarthritis
OP: osteoporosis
PI: national principal investigator
RA: rheumatoid arthritis
RMD: rheumatic and musculoskeletal diseases
SG: Steering Group
SpA: spondyloarthritis
UEMS: European Union of Medical Specialists
US: ultrasound
