# Erratum to: Rheumatology training experience across Europe: analysis of core competences

**DOI:** 10.1186/s13075-016-1199-3

**Published:** 2016-12-19

**Authors:** Francisca Sivera, Sofia Ramiro, Nada Cikes, Maurizio Cutolo, Maxime Dougados, Laure Gossec, Tore K. Kvien, Ingrid E. Lundberg, Peter Mandl, Arumugam Moorthy, Sonia Panchal, José A. P. da Silva, Johannes W. Bijlsma, the Working Group on Training in Rheumatology across Europe

**Affiliations:** 1 Department Reumatologia, Hospital General Universitario de Elda, ctra Sax s/n, Elda, Alicante, 03600 Spain; 2 Leiden University Medical Center, Leiden, The Netherlands; 3 University of Zagreb School of Medicine, University Hospital Centre, Zagreb, Croatia; 4 Research Laboratories and Academic Division of Clinical Rheumatology, Postgraduate School on Rheumatology, Department of Internal Medicine University of Genova, Genova, Italy; 5 Université Paris Descartes University, Department of Rheumatology, Hôpital Cochin, Assistance Publique-Hôpitaux de Paris; INSERM (U1153): Epidemiologie Clinique et Biostatistiques, PRES Sorbonne Paris-Cité, Paris, France; 6 Sorbonne Universités, UPMC Univ Paris 06, Institut Pierre Louis d’Epidémiologie et de Santé Publique; AP-HP, Pitié Salpêtrière Hospital, Department of Rheumatology, F-75013 Paris, France; 7 Department of Rheumatology, Diakonhjemmet Hospital, Oslo, Norway; 8 Rheumatology Unit, Department of Medicine, Karolinska University Hospital, Solna, Sweden; 9 Karolinska Institutet, Stockholm, Sweden; 10 Divison of Rheumatology, Medical University of Vienna, Vienna, Austria; 11 University Hospitals of Leicester, Leicester, UK; 12 University Hospitals of Leicester NHS Trust, Leicester, UK; 13 Centro Hospitalar e Universitário de Coimbra, Faculdade de Medicina da Universidade de Coimbra, Coimbra, Portugal; 14 University Medical Center Utrecht, Utrecht, The Netherlands

## Erratum

Unfortunately, after publication of this article [1], it was noticed that the affiliation for Peter Mandl was incorrect. The correct affiliation is ‘Divison of Rheumatology, Medical University of Vienna, Vienna, Austria’. This can be seen in the corrected author details below.
