# Supplementary material for: Rheumatology training experience across Europe: analysis of core competences
Source: Arthritis Res Ther. 2016 Sep 23;18:213. doi: 10.1186/s13075-016-1114-y (PMC5035447; doi:10.1186/s13075-016-1114-y)
Supplement: Additional file 1: — Survey of the project “Assessment of training for Rheumatology fellows across Europe”. (DOCX 28 kb) [file 13075_2016_1114_MOESM1_ESM.docx]

Additional file 1: Survey of the project "Assessment of training for Rheumatology fellows across Europe"

**Introductory paragraph**

This survey is part of an ongoing project that aims to assess the differences in rheumatology training across European countries.

Throughout the survey you will be asked about some aspects of your rheumatology training.

If you are a qualified rheumatologist, please reflect any training performed during your official rheumatology training program, but for the purpose of this survey please ignore any additional training done after the completion of the official training program and becoming a certified rheumatologist.

If you are still a trainee, please answer based on your reasonable expectations of what you will achieve by the end of your rheumatology training program, taking into account what you have acquired so far during your training and the usual standards in your country or training program.

It will take approximately 15 minutes to complete survey. Thank you for taking the time to help us understand your rheumatology training!

**Demographics**

*Please answer these questions regarding yourself and the country in which you have trained (irrespective of the country where you are currently working in).*

1. In which country did you train? (drop-down list)
2. What is your age: less than or equal to 25, 26-30; 31-35, 36-40, more than 40.
3. What is your gender? (Single-choice question: Male/Female)
4. Are you a trainee or a certified rheumatologist? (Single-choice question: Trainee in Rheumatology (i.e. your training is not yet completed) /Specialist in Rheumatology (training completed, whether currently working as rheumatologist or not)
5. What is your current year of training?

If you are already a specialist in rheumatology, please answer not applicable. If you are a trainee, please consider as the first year, the beginning of the rheumatology training program. Please bear in mind that in some countries the rheumatology training program will include training in internal medicine while in others this will be performed before the start of the rheumatology training program. (year 1, year 2, year 3, year 4, year 5, year 6, year 7, not applicable)

1. How many years ago did you finish your rheumatology training?

If you are a trainee, please answer not applicable. (1 year, 2 years, 3 years, 4 years, 5 years, >5 years, not applicable)

**Achievement of competences**

**In this section we are going to ask you about some of the competences you achieved during your training period in rheumatology and to what degree. If you are now a fully certified rheumatologist, please think back to the time when you had just finished your training and judge your ability at that time point. If you are still a trainee, please estimate the ability you expect to achieve by the end of your training taking into account your current competence and reasonable expectations for the time of training you have left.**

1. By the end of your training, how would you judge your ability to perform the following competences on a 0-10 scale (where 0 means "Unable to do" and 10 means "Fully able to do independently")?

🞏 0 🞏 1 🞏 2 🞏 3 🞏 4 🞏 5 🞏 6 🞏 7 🞏 8 🞏 9 🞏 10

Unable to do Fully able to do

independently

- perform a clinical examination of the musculoskeletal (MSK) system
- detect synovitis of MCP and PIP joints
- perform the initial diagnostic approach and management plan for a patient with a single swollen joint (possible monoarthritis)
- interpret laboratory tests (RF, antiCCP/ACPAs, HLAB27, ANA)
- manage a patient with osteoarthritis (OA)
- manage a patient with gout
- manage a patient with early rheumatoid arthritis / undifferentiated arthritis
- manage a patient with spondyloarthritis (SpA)
- manage a patient with an autoimmune connective tissue disease
- manage a patient with a systemic vasculitis
- manage a patient with osteoporosis
- initiate and monitor therapy with a biologic DMARD
- use, apply and interpret measures of disease activity commonly used in rheumatic diseases
- perform a knee aspiration
- identify monosodium urate (MSU) and calcium pyrophosphate (CPPD) crystals on polarized microscopy
- interpret a conventional hand X-ray
- ability to perform a musculoskeletal (MSK) ultrasound
- work within a multidisciplinary team (a team of diverse specialists systematically working together and not simply consulting another specialty)
- interpret the results of a scientific publication
- ability to present (i.e. within the department or hospital, local meetings, congresses...)
- ability to communicate (i.e. communication with patients, families and colleagues)

**Formal education**

**In this section we are asking whether you received formal education in some specific areas and competences. Formal education does not mean that you have seen, managed or discussed specific patients. Rather, it includes courses, lectures, specific sessions, etc. that you participated in during your rheumatology training period (both as part of your training program or as optional courses).**

1. During your rheumatology training, did you receive formal education in the following competences? This does not mean that you have seen, managed or discussed patients with these complaints or that you can treat patients with these diseases. Rather we are asking if you have received formal education such as courses, lectures, etc that you participated in during your rheumatology training period (both as part of your training program or as optional courses). Please disregard any education performed after after the completion of the official training program and becoming a certified rheumatologist.

Yes/No

- perform a clinical examination of the musculoskeletal (MSK) system
- detection of synovitis of MCP and PIP joints
- perform the initial diagnostic approach and management plan for a patient with a single swollen joint (possible monoarthritis)
- interpret laboratory tests (RF, antiCCP/ACPAs, HLAB27, ANA)
- manage a patient with osteoarthritis (OA)
- manage a patient with gout
- manage a patient with early rheumatoid arthritis / undifferentiated arthritis
- manage a patient with spondyloarthritis (SpA)
- manage a patient with an autoimmune connective tissue disease
- manage a patient with a systemic vasculitis
- manage a patient with osteoporosis
- initiate and monitor therapy with a biologic DMARD
- use, apply and interpret measures of disease activity commonly used in rheumatic diseases

1. During your rheumatology training, did you receive formal education or training in the following skills? This does not mean that you have performed these procedures independently, but rather if you have received training such as hands-on/practical courses, one-on-one tutoring, etc. that you participated in during your rheumatology training period (both as part of your training program or as optional courses)

Yes/No

- perform a knee aspiration
- identify MSU and CPPD crystals on polarized microscopy
- interpret a conventional hand X-ray
- perform an MSK US

1. During your rheumatology training, did you receive formal education or training in the following competences? This could include courses, lectures, journal clubs, etc. that you participated in during your rheumatology training period (both as part of your training program or as optional courses**)**

Yes/No

- interpret the results of a scientific publication
- presentation skills (i.e. presentations performed at the department or hospital, local meetings, congresses..)
- communication skills (i.e. communicating with patients, families and colleagues)

**Patient management**

**In this section we are asking you to estimate how many patients with these specific complaints you have managed during your rheumatology training. For the purpose of this survey managing a patient implies that you had some degree of responsibility in their treatment and/or follow-up**.

1. Please provide the approximate number of patients with the following conditions that you managed during your rheumatology training. Managing in this context implies that you had some degree of responsibility in the treatment and/or follow-up of these patients. We are aware of the level of difficulty in making such an estimation so please make the best educated guess possible. Ranges: 0; 1-10; 11-50; 51-100: 101-150; >150.

- patients presenting with a single swollen joint (possible monoarthritis)
- patients with osteoarthritis (OA)
- patients with gout
- patients with early rheumatoid arthritis / undifferentiated arthritis
- patients with spondyloarthritis (SpA)
- patients with an autoimmune connective tissue disease
- patients with a systemic vasculitis
- patients with osteoporosis
- patients that you initiated and/or monitored therapy with a biologic DMARD

1. By the end of your training, did you work quasi-independently in an outpatient clinic in which you took most of the decisions independently with only minor (direct) supervision?

Yes/No

1. During your rheumatology training, did you have some degree of responsibility in initiating therapy and monitoring patients treated with a biologic DMARD?

Yes/No

1. During your rheumatology training, did you regularly (or a least frequently) apply measures of disease activity commonly used in rheumatic disease?

Yes/No

**Skills and procedures**

**In this section we ask you to estimate how many technical procedures you performed during your rheumatology training.**

1. Please provide an approximate number of procedures that you performed during your rheumatology training.

Ranges: 0; 1-10; 11-50; 51-100: 101-150; >150

- knee aspiration
- identification of MSU and CPPD crystals on polarized microscopy
- interpretation of a conventional hand X-ray
- perform a MSK ultrasound

1. Please provide an approximate number of presentations that you gave during your rheumatology training. For the number given, please consider presentations at local, national or international congresses, within the department, at other meetings, for other specialists, etc.

Ranges: 0; 1-10; 11-50; 51-100: 101-150; >150

1. During your rheumatology training, did you participate in a research project that has been published in an indexed peer-reviewed journal where you have been 1st or 2nd author?

Yes/No

**Competence assessment**

**In this section, we want to know whether each of the following competences is the object of formal assessment in your country or training program. These assessments might be performed during your training or at the end of your training period (i.e. final exam). We are not asking if you are able to perform these competences, but rather if you were formally assessed in the following competences.**

**Please note: Non-structured assessments by your supervisor should not be considered as formal assessment. Rather we are asking about exams, practical evaluations or other types of formal assessment that must to be undertaken before you become a qualified rheumatologist.**

1. During your rheumatology training, was your ability in the following competences formally assessed? This does not include non-structured assessments by your supervisor, but rather exams, practical evaluations, or other forms of formal assessment.

Yes/No

- performing a clinical examination of the musculoskeletal (MSK) system
- detection of synovitis of MCP and PIP joints
- performing the initial diagnostic approach and management plan for a patient with a single swollen joint (possible monoarthritis)
- interpreting laboratory tests (RF, antiCCP/ACPAs, HLAB27, ANA)
- managing a patient with osteoarthritis (OA)
- managing a patient with gout
- managing a patient with early rheumatoid arthritis / undifferentiated arthritis
- managing a patient with spondyloarthritis (SpA)
- managing a patient with an autoimmune connective tissue disease
- managing a patient with a systemic vasculitis
- managing a patient with osteoporosis
- initiating and monitoring therapy with a biologic DMARD
- using, applying and interpreting measures of disease activity commonly used in rheumatic diseases
- performing a knee aspiration
- identifying MSU and CPPD crystals on polarized microscopy
- interpreting a conventional hand X-ray
- performing a MSK ultrasound
- working within a multidisciplinary team
- interpreting the results of a scientific publication
- presentation skills (i.e. presentations performed at the department or hospital, local meetings, congresses..)
- communication skills (i.e. communicating with patients, families and colleagues)

1. Do you keep a log-book/portfolio (i.e. place in which the activities of training should be recorded)?

Yes/No

**Assessment opinion**

**We would now like to know your opinion on what a rheumatology training program should include and how competences should be assessed.**

1. Which of the following assessments do you think should be undertaken at the end of or during your training before receiving your certification in rheumatology? (You can select several options, but please select only those that in your opinion should always be performed, (ie in every trainee)

Yes/No

- Written theoretical exam
- Oral theoretical exam
- Practical exam (i.e. history taking and/or physical examination and/or clinical reasoning including case-based discussions)
- Evaluation and discussion of the clinical curriculum achieved by the trainee
- Evaluation and discussion of the general curriculum (research included) achieved by the trainee
- Assessment of generic skills (communication, leadership, team working, reliability, etc, across the domains of Good Medical Practice)
- Evaluation of procedural skills (to assess the performance of a trainee in undertaking a practical procedure, against a structured checklist)
- Assessment of the trainee by a patient (to address issues, including behavior of the doctor and effectiveness of the consultation, interpersonal skills, communication skills and professionalism)
- Teaching observation (to evaluate the trainee’s competence in teaching)
- EULAR on-line course (including final exam)
- None, certification should be automatically attributed at the end of the period of training without a formal assessment
- Other? Specify _____________________

1. In order to gain further insight into what you think EVERY rheumatologist should know and should be able to do, could you list which competences you think are essential for physicians specializing in rheumatology. When answering, we would like you to consider both rheumatology-specific competences (For example, "a rheumatologist should be able aspirate all peripheral joints") or generic competences (For example, "a rheumatologist should be able to construct an appropriate relationship with patients and families"). (No word limit)

(Open-ended comment box).

You have now finished the survey.

Thank you for your time!

Please help us disseminate this survey! We need wide participation from each country to achieve relevant results, so any additional participation is very much valued!

Feel free to send the following link to your contacts (trainees or rheumatologists within 5 years of training): https://www.surveymonkey.com/s/5LTVPSJ
