# Supplementary material for: Rheumatology training experience across Europe: analysis of core competences
Source: Arthritis Res Ther. 2016 Sep 23;18:213. doi: 10.1186/s13075-016-1114-y (PMC5035447; doi:10.1186/s13075-016-1114-y)
Supplement: Additional file 2: Table S1. — Response numbers and rate per country. (DOCX 16 kb) [file 13075_2016_1114_MOESM2_ESM.docx]

Additional file 2:

Table: Response numbers and rate per country

| **Country** | **Number of responses per country** | **Percentage of total responses (number of responses from country/total number of responses received)*100** | **Target population per country** | **Percentage of responses compared to target population (per country)§** |
| --- | --- | --- | --- | --- |
| Albania | 9 | 0.8 | 39 | 23.1 |
| Armenia | 2 | 0.2 | 21 | 9.5 |
| Austria | 17 | 1.6 | 58 | 29.3 |
| Belarus | 3 | 0.3 | 121 | 2.5 |
| Belgium | 18 | 1.7 | 56 | 32.1 |
| Bosnia | 7 | 0.7 | 7 | 100.0 |
| Bulgaria | 8 | 0.7 | 71 | 11.3 |
| Croatia | 7 | 0.7 | 24 | 29.2 |
| Czech Republic | 33 | 3.1 | 40 | 82.5 |
| Denmark | 86 | 8.0 | 171 | 50.3 |
| Estonia | 8 | 0.7 | 8 | 100.0 |
| Finland | 14 | 1.3 | 43 | 32.6 |
| France | 140 | 13.0 | 225 | 62.2 |
| Georgia | 7 | 0.7 | 20 | 35.0 |
| Germany | 20 | 1.9 | 392 | 5.1 |
| Greece | 43 | 4.0 | 108 | 39.8 |
| Hungary | 39 | 3.6 | 56 | 69.6 |
| Ireland | 18 | 1.7 | 24 | 75.0 |
| Israel | 21 | 2.0 | 60 | 35.0 |
| Italy | 16 | 1.5 | 280 | 5.7 |
| Lebanon | 7 | 0.7 | 15 | 20.0 |
| Latvia | 3 | 0.3 | 14 | 50.0 |
| Lithuania | 9 | 0.7 | 21 | 33.3 |
| Macedonia | 5 | 0.5 | 21 | 23.8 |
| Malta | 2 | 0.2 | 5 | 40.0 |
| Moldova | 2 | 0.2 | 26 | 7.7 |
| Netherlands | 26 | 2.4 | 184 | 14.1 |
| Norway | 47 | 4.4 | 207 | 22.7 |
| Poland | 61 | 5.7 | 440 | 13.9 |
| Portugal | 41 | 3.8 | 90 | 45.6 |
| Romania | 62 | 5.8 | 180 | 34.4 |
| Russia | 26 | 2.4 | 166 | 15.7 |
| Serbia | 14 | 1.3 | 70 | 20.0 |
| Slovakia | 16 | 1.5 | 36 | 44.4 |
| Slovenia | 11 | 1.0 | 17 | 64.7 |
| Spain | 84 | 7.8 | 440 | 19.1 |
| Sweden | 22 | 2.0 | 144 | 15.3 |
| Switzerland | 26 | 2.4 | 135 | 19.3 |
| Turkey (through internal medicine)* | 39 | 3.6 | 64 | 60.9 |
| Turkey (through physical therapy)* | 12 | 1.1 | 60 | 20.0 |
| United Kingdom | 47 | 4.4 | 240 | 19.6 |
| Ukraine | 3 | 0.3 | 84 | 3.6 |

* In Turkey, training in rheumatology can take place through two routes, through internal medicine or through physical therapy

§ Target population was obtained by multiplying the number of trainees who started the training in a given country in the year before the survey (as determined by the national PI) times the number of years of the rheumatology-specific period of training plus five years (in order to include rheumatologists certified in the past five years).
