# Supplementary material for: Rheumatology training experience across Europe: analysis of core competences
Source: Arthritis Res Ther. 2016 Sep 23;18:213. doi: 10.1186/s13075-016-1114-y (PMC5035447; doi:10.1186/s13075-016-1114-y)
Supplement: Additional file 3: Table S2. — Number and percentage of respondents with very low self-reported ability per competence. (DOCX 14 kb) [file 13075_2016_1114_MOESM3_ESM.docx]

Additional file 3

Table: Number and percentage of respondents with a very low self-reported ability per competence

|  | **Very low self-reported ability (NRS<3)** |
| --- | --- |
| **Musculoskeletal exam** | 3 (0.3%) |
| **Detect synovitis** | 4 (0.4%) |
| **Monoarthritis** | 3 (0.3%) |
| **Lab tests interpretation** | 2 (0.2%) |
| **Osteoarthritis*** | 7 (0.7%) |
| **Gout*** | 3 (0.3%) |
| **Early Rheumatoid arthritis*** | 3 (0.3%) |
| **Spondyloarthritis*** | 2 (0.2%) |
| **Autoimmune connective tissue diseases*** | 17 (1.6%) |
| **Vasculitis*** | 37 (3.5%) |
| **Osteoporosis*** | 7 (0.7%) |
| **bDMARD*** | 18 (1.7%) |
| **Disease activity measures** | 10 (0.9%) |
| **Knee aspiration** | 35 (3.3%) |
| **Crystals identification** | 285 (26.6%) |
| **X-ray** | 18 (1.7%) |
| **Ultrasound** | 252 (23.8%) |
| **Multidisciplinary team** | 47 (4.4%) |
| **Interpret published paper** | 26 (2.4%) |
| **Presentation** | 28 (2.6%) |
| **Communication** | 8 (0.7%) |

NRS: numerical rating scale

* These competences refer to the management of a patient with the given disease or treatment
