# Supplementary material for: Rheumatology training experience across Europe: analysis of core competences
Source: Arthritis Res Ther. 2016 Sep 23;18:213. doi: 10.1186/s13075-016-1114-y (PMC5035447; doi:10.1186/s13075-016-1114-y)
Supplement: Additional file 4: Table S3. — Comparison of self-reported ability in each competence in respondents managing ≤10 patients and those managing >10 patients in the corresponding competence during their training. (DOCX 15 kb) [file 13075_2016_1114_MOESM4_ESM.docx]

Additional file 4

Table: Comparison of self-reported ability in each competence in respondents managing ≤ 10 patients and those managing > 10 patients in the corresponding competence during their training

|  | **Self-reported ability in respondents managing**  **≤ 10 patients (mean (SD))** | **Self-reported ability in respondents managing**  **> 10 patients (mean (SD))** | **p-value** |
| --- | --- | --- | --- |
| **MSK exam** |  |  |  |
| **Detect synovitis** |  |  |  |
| **Monoarthritis** | 8.2 (1.9) | 9.2 (1.2) | <0.0001 |
| **Lab tests interpretation** |  |  |  |
| **Osteoarthritis*** | 7.5 (2.69) | 9.0 (1.4) | <0.0001 |
| **Gout*** | 8.1 (2.1) | 9.3 (1.1) | <0.0001 |
| **Early Rheumatoid arthritis*** | 7.9 (2.2) | 9.2 (1.2) | <0.0001 |
| **Spondyloarthritis*** | 8.1 (2.1) | 9.1 (1.3) | <0.0001 |
| **Autoimmune connective tissue diseases*** | 6.6 (2.2) | 8.4 (1.6) | <0.0001 |
| **Vasculitis*** | 6.6 (2.4) | 8.2 (1.7) | <0.0001 |
| **Osteoporosis*** | 7.4 (2.2) | 8.8 (1.5) | <0.0001 |
| **bDMARD*** | 7.2 (2.7) | 9.2 (1.3) | <0.0001 |
| **Disease activity measures** |  |  |  |
|  |  |  |  |
| **Knee aspiration** | 6.5 (3.2) | 9.5 (1.0) | <0.0001 |
| **Crystals identification** | 4.1 (3.6) | 9.1 (1.5) | <0.0001 |
| **X-ray** | 5.9 (2.5) | 8.5 (1.6) | <0.0001 |
| **Ultrasound** | 2.9 (3.0) | 7.7 (2.1) | <0.0001 |
|  |  |  |  |
| **Multidisciplinary team** |  |  |  |
| **Interpret published paper** |  |  |  |
| **Presentation** | 7.3 (2.4) | 8.7 (1.6) | <0.0001 |
| **Communication** |  |  |  |

NS: not-significant

* These competences refer to the management of a patient with the given disease or treatment
