# Supplementary material for: Rheumatology training experience across Europe: analysis of core competences
Source: Arthritis Res Ther. 2016 Sep 23;18:213. doi: 10.1186/s13075-016-1114-y (PMC5035447; doi:10.1186/s13075-016-1114-y)
Supplement: Additional file 5: Table S4. — Comparison of self-reported ability in each competence in respondents with and without assessment in the corresponding competence. (DOCX 15 kb) [file 13075_2016_1114_MOESM5_ESM.docx]

Additional file 5

Table: Comparison of self-reported ability in each competence in respondents with and without assessment in the corresponding competence

|  | **Self-reported ability in respondents who were not assessed (mean (SD))** | **Self-reported ability in respondents who were assessed (mean (SD))** | **p-value** |
| --- | --- | --- | --- |
| **MSK exam** | 8.8 (1.6) | 9.2 (1.3) | 0.0001 |
| **Detect synovitis** | 9.0 (1.5) | 9.1 (1.3) | NS |
| **Monoarthritis** | 9.1 (1.3) | 9.2 (1.4) | NS |
| **Lab tests interpretation** | 9.4 (1.1) | 9.4 (1.0) | NS |
| **Osteoarthritis*** | 8.6 (1.7) | 9.2 (1.3) | <0.0001 |
| **Gout*** | 8.9 (1.5) | 9.3 (1.2) | <0.0001 |
| **Early Rheumatoid arthritis*** | 9.0 (1.5) | 9.1 (1.3) | NS |
| **Spondyloarthritis*** | 8.9 (1.4) | 9.1 (1.3) | NS |
| **Autoimmune connective tissue diseases*** | 7.6 (2.1) | 8.4 (1.7) | <0.0001 |
| **Vasculitis*** | 7.1 (2.3) | 7.8 (1.9) | <0.0001 |
| **Osteoporosis*** | 8.4 (1.8) | 8.9 (1.4) | <0.0001 |
| **bDMARD*** | 8.8 (1.9) | 9.0 (1.6) | NS |
| **Disease activity measures** | 8.8 (1.6) | 9.0 (1.4) | 0.006 |
|  |  |  |  |
| **Knee aspiration** | 8.7 (2.4) | 9.3 (1.5) | NS |
| **Crystals identification** | 5.2 (3.9) | 8.1 (2.7) | <0.0001 |
| **X-ray** | 7.9 (2.0) | 8.5 (1.7) | <0.0001 |
| **Ultrasound** | 4.6 (3.6) | 7.5 (2.5) | <0.0001 |
|  |  |  |  |
| **Multidisciplinary team** |  |  |  |
| **Interpret published paper** | 7.7 (2.1) | 8.2 (1.9) | <0.0001 |
| **Presentation** | 7.9 (2.2) | 8.4 (8.2) | 0.0037 |
| **Communication** | 8.9 (1.5) | 9.0 (1.4) | NS |

NS: not significant

* These competences refer to the management of a patient with the given disease or treatment
