# Supplementary material for: Rheumatology training experience across Europe: analysis of core competences
Source: Arthritis Res Ther. 2016 Sep 23;18:213. doi: 10.1186/s13075-016-1114-y (PMC5035447; doi:10.1186/s13075-016-1114-y)
Supplement: Additional file 6: Table S5. — Comparison of self-reported ability in each competence in respondents with a long and short length of training (previous training in internal medicine plus rheumatology training program). (DOCX 15 kb) [file 13075_2016_1114_MOESM6_ESM.docx]

Additional file 6

Table: Comparison of self-reported ability in each competence in respondents with a long and short length of training (previous training in internal medicine plus rheumatology training program)

|  | **Self-reported ability in respondents with a long length of training^¥^ (mean (SD))** | **Self-reported ability in respondents with a short length of training^¥^ (mean (SD))** | **p-value** |
| --- | --- | --- | --- |
| **MSK exam** | 9.1 (1.4) | 8.8 (1.5) | <0.0001 |
| **Detect synovitis** | 9.3 (1.2) | 8.8 (1.6) | <0.0001 |
| **Monoarthritis** | 9.3 (1.3) | 8.9 (1.4) | <0.0001 |
| **Lab tests interpretation** | 9.5 (1.0) | 9.3 (1.2) | <0.0001 |
| **Osteoarthritis*** | 9.0 (1.6) | 8.8 (1.6) | <0.001 |
| **Gout*** | 9.3 (1.2) | 8.9 (1.5) | <0.0001 |
| **Early Rheumatoid arthritis*** | 9.3 (1.2) | 8.8 (1.5) | <0.0001 |
| **Spondyloarthritis*** | 9.2 (1.2) | 8.7 (1.5) | <0.0001 |
| **Autoimmune connective tissue diseases*** | 8.3 (1.6) | 7.7 (2.1) | <0.0001 |
| **Vasculitis*** | 7.4 (2.2) | 7.5 (2.1) | NS |
| **Osteoporosis*** | 8.8 (1.6) | 8.6 (1.7) | <0.05 |
| **bDMARD*** | 9.1 (1.6) | 8.6 (1.9) | <0.0001 |
| **Disease activity measures** | 9.0 (1.5) | 8.8 (1.6) | <0.01 |
|  |  |  |  |
| **Knee aspiration** | 9.1 (2.0) | 8,8 (2.1) | <0.0001 |
| **Crystals identification** | 6.8 (3.7) | 5.3 (3.8) | <0.0001 |
| **X-ray** | 8.2 (1.9) | 8.1 (1.9) | <0.05 |
| **Ultrasound** | 6.1 (3.5) | 5.8 (3.3) | <0.01 |
|  |  |  |  |
| **Multidisciplinary team** | 8.4 (2.2) | 7.8 (2.3) | <0.0001 |
| **Interpret published paper** | 8.2 (1.9) | 7.5 (2.2) | <0.0001 |
| **Presentation** | 8.4 (2.0) | 7.7 (2.2) | <0.0001 |
| **Communication** | 9.2 (1.3) | 8.6 (1.6) | <0.0001 |

**^¥^** Training length was considered length of prior training in internal medicine + length of rheumatology training program. Long training was defined as equal to or greater than the median length. Short training was defined as less than the median length.

* These competences refer to the management of a patient with the given disease or treatment
