# Supplementary material for: Rheumatology training experience across Europe: analysis of core competences
Source: Arthritis Res Ther. 2016 Sep 23;18:213. doi: 10.1186/s13075-016-1114-y (PMC5035447; doi:10.1186/s13075-016-1114-y)
Supplement: Additional file 8: Table S7. — Self-reported ability per individual country. (DOCX 23 kb) [file 13075_2016_1114_MOESM8_ESM.docx]

Additional file 8

Table: Self-reported ability per individual country

| **Country** | **MSK exam** | **Detect synovitis** | **Monoarthritis** | **Lab tests interpretation** | **OA*** | **Gout*** | **Early RA*** | **SpA*** | **Autoimmune CTD*** | **Vasculitis*** | **Osteoporosis*** | **bDMARD*** | **Disease activity measures** | **Knee aspiration** | **Crystals identification** | **X-ray** | **Ultrasound** | **Multidisciplinary team** | **Interpret published paper** | **Presentation** | **Communication** |
| --- | --- | --- | --- | --- | --- | --- | --- | --- | --- | --- | --- | --- | --- | --- | --- | --- | --- | --- | --- | --- | --- |
| Albania | 8.1 (3.2) | 8.1 (3.2) | 7.7 (3.0) | 8.3 (3.2) | 7.7 (3.2) | 8.0 (3.2) | 7.0 (2.9) | 7.0 (3.0) | 6.2 (2.9) | 5.9 (2.9) | 8.4 (3.2) | 6.2 (3.2) | 6.7 (3.3) | 4.7 (3.4) | 0.1 (0.3) | 7.4 (3.5) | 0.0 (0) | 4.9 (4.1) | 6.4 (3.0) | 7.3 (3.5) | 8.3 (3.3) |
| Armenia | 9.0 (0) | 9.0 (1.4) | 9.0 (0) | 9.5 (0.7) | 9.5 (0.7) | 8.0 (1.4) | 7.5 (0.7) | 8.5 (0.7) | 5.5 (3.5) | 5.0 (2.8) | 9.5 (0.7) | 7.5 (2.1) | 8.0 (1.4) | 9.0 (0) | 5.0 (7.1) | 8.5 (0.7) | 2.0 (2.8) | 8.5 (2.1) | 8.5 (2.1) | 9.0 (1.4) | 9.5 (0.7) |
| Austria | 7.8 (2.1) | 9.5 (0.9) | 9.5 (0.9) | 9.8 (0.4) | 9.1 (1.5) | 9.4 (1.1) | 9.5 (0.7) | 9.8 (0.6) | 8.6 (1.5) | 8.4 (1.8) | 9.0 (1.2) | 9.8 (0.7) | 9.5 (0.8) | 8.8 (2.5) | 7.2 (3.5) | 7.6 (2.1) | 6.7 (3.8) | 8.5 (1.7) | 8.8 (1.7) | 8.6 (2.5) | 9.47 (0.7) |
| Belarus | 10.0 (0) | 10.0 (0) | 10.0 (0) | 10.0 (0) | 10.0 (0) | 10.0 (0) | 10.0 (0) | 10.0 (0) | 10.0 (0) | 10.0 (0) | 9.3 (1.2) | 10.0 (0) | 9.7 (0.6) | 6.3 (1.5) | 0.7 (1.2) | 9.3 (0.6) | 0.7 (1.2) | 9.3 (0.6) | 9.7 (0.6) | 9.3 (0.6) | 9.7 (0.6) |
| Belgium | 8.8 (1.5) | 8.9 (0.9) | 9.4 (0.8) | 9.3 (0.8) | 9.0 (1.1) | 9.3 (0.8) | 9.0 (0.8) | 9.0 (1.0) | 7.6 (1.4) | 7.3 (1.5) | 8.7 (1.7) | 9.2 (0.7) | 8.9 (0.9) | 9.7 (0.8) | 4.7 (4.2) | 8.3 (1.4) | 4.9 (3.6) | 8.7 (1.0) | 8.2 (1.2) | 8.4 (1.3) | 8.9 (1.1) |
| Bosnia | 10.0 (0) | 10.0 (0) | 9.9 (0.4) | 10.0 (0) | 10.0 (0) | 9.7 (0.5) | 9.7 (0.5) | 10.0 (0) | 9.1 (0.7) | 7.3 (2.3) | 9.1 (1.1) | 8.1 (1.2) | 9.3 (1.0) | 7.1 (3.4) | 2.7 (2.6) | 8.6 (0.8) | 0.7 (1.9) | 9.4 (1.0) | 8.7 (1.8) | 9.1 (0.9) | 9.6 (0.8) |
| Bulgaria | 8.8 (2.1) | 9.4 (0.7) | 9.5 (0.8) | 9.8 (0.7) | 9.6 (0.7) | 9.8 (0.7) | 9.6 (07) | 9.8 (0.7) | 8.9 (1.6) | 8.6 (1.9) | 9.5 (0.8) | 8.3 (1.3) | 9.3 (1.0) | 8.3 (3.0) | 6.9 (1.9) | 8.9 (1.4) | 6.4 (3.7) | 7.1 (2.6) | 6.3 (3.3) | 7.0 (3.0) | 8.6 (1.4) |
| Croatia | 7.9 (2.0) | 9.0 (1.4) | 8.6 (1.4) | 9.4 (1.0) | 8.9 (1.9) | 9.0 (1.2) | 9.1 (0.9) | 9.0 (1.2) | 8.1 (1.4) | 7.6 (1.8) | 7.9 (1.7) | 8.0 (1.4) | 8.6 (1.4) | 7.4 (3.5) | 3.0 (2.7) | 7.1 (2.3) | 5.4 (3.5) | 8.1 (1.4) | 8.1 (1.8) | 8.3 (1.4) | 9.1 (0.7) |
| Czech Republic | 8.8 (1.5) | 8.6 (1.7) | 8.6 (1.9) | 9.0 (1.3) | 8.9 (1.5) | 9.1 (1.3) | 8.8 (1.9) | 9.0 (1.4) | 8.0 (2.2) | 7.6 (2.1) | 8.1 (2.0) | 7.9 (3.2) | 8.4 (2.0) | 9.0 (1.5) | 5.0 (3.9) | 6.6 (2.2) | 5.1 (3.4) | 7.2 (2.9) | 6.5 (3.5) | 6.7 (3.2) | 8.5 (2.3) |
| Denmark | 9.4 (1.0) | 9.7 (0.7) | 9.9 (0.4) | 9.6 (0.7) | 9.5 (0.8) | 9.7 (0.6) | 9.7 (0.7) | 9.5 (0.9) | 7.9 (2.0) | 7.7 (2.1) | 9.0 (1.3) | 9.4 (1.0) | 9.4 (1.0) | 9.9 (0.3) | 8.8 (2.0) | 7.9 (2.3) | 8.6 (1.5) | 8.9 (1.9) | 8.7 (1.5) | 9.0 (1.2) | 9.6 (0.6) |
| Estonia | 9.9 (0.4) | 9.6 (0.5) | 9.8 (0.7) | 9.5 (1.1) | 8.6 (3.5) | 8.4 (3.5) | 9.6 (0.7) | 9.6 (0.7) | 8.6 (0.9) | 8.4 (1.3) | 9.0 (2.5) | 9.4 (1.4) | 9.8 (0.7) | 8.9 (1.4) | 3.3 (3.7) | 9.1 (1.8) | 5.4 (4.1) | 7.1 (2.4) | 8.9 (1.9) | 9.0 (0.9) | 9.6 (1.1) |
| Finland | 8.8 (0.9) | 9.0 (1.0) | 9.1 (1.1) | 9.3 (0.6) | 8.1 (1.4) | 9.1 (0.8) | 9.1 (1.1) | 8.9 (1.2) | 8.6 (1.1) | 8.0 (1.1) | 8.8 (1.1) | 9.2 (0.8) | 8.9 (1.0) | 9.8 (0.4) | 3.9 (4.1) | 8.6 (1.3) | 8.4 (1.8) | 8.1 (1.8) | 8.2 (1.2) | 8.4 (1.7) | 8.9 (1.5) |
| France | 8.2 (1.6) | 8.4 (1.4) | 8.5 (1.4) | 9.0 (1.1) | 8.1 (1.6) | 8.3 (1.4) | 8.1 (1.7) | 8.2 (1.5) | 6.6 (1.8) | 5.7 (2.0) | 8.0 (1.6) | 8.5 (1.6) | 8.6 (1.5) | 9.4 (1.1) | 4.8 (3.7) | 7.9 (1.7) | 6.2 (3.1) | 7.7 (2.0) | 6.9 (1.7) | 6.9 (1.9) | 7.9 (1.7) |
| Georgia | 8.1 (1.7) | 8.4 (1.6) | 8.6 (1.7) | 9.0 (1.8) | 9.0 (1.8) | 8.9 (1.9) | 9.1 (1.9) | 8.4 (2.1) | 8.1 (2.0) | 7.1 (2.4) | 8.9 (1.9) | 8.0 (1.8) | 7.9 (2.2) | 6.1 (3.9) | 5.7 (3.7) | 8.4 (1.7) | 6.0 (2.5) | 8.6 (1.8) | 8.7 (1.8) | 8.3 (1.8) | 9.3 (1.9) |
| Germany | 8.9 (1.8) | 9.6 (0.8) | 9.2 (1.3) | 9.5 (0.8) | 8.9 (2.0) | 8.9 (1.7) | 9.2 (1.3) | 9.3 (1.3) | 8.5 (1.5) | 7.6 (2.2) | 8.3 (2.0) | 9.6 (0.6) | 9.1 (1.9) | 8.8 (2.1) | 6.2 (3.5) | 8.1 (2.4) | 8.2 (2.4) | 8.3 (2.4) | 8.4 (1.9) | 8.4 (1.8) | 9.3 (1.4) |
| Greece | 8.8 (1.7) | 8.9 (1.3) | 8.7 (1.5) | 9.2 (1.1) | 8.7 (1.3) | 9.2 (1.0) | 8.8 (1.5) | 8.8 (1.3) | 8.3 (1.4) | 7.7 (1.8) | 8.6 (1.4) | 8.9 (1.6) | 8.2 (1.9) | 9.2 (1.4) | 8.2 (2.4) | 8.1 (2.1) | 3.0 (3.2) | 7.1 (2.6) | 7.2 (2.2) | 7.5 (2.0) | 8.8 (1.3) |
| Hungary | 9.1 (1.2) | 9.1 (1.0) | 8.7 (1.5) | 9.1 (1.6) | 9.0 (1.5) | 8.6 (1.9) | 8.7 (1.7) | 8.5 (1.7) | 7.2 (2.3) | 6.0 (2.9) | 8.7 (1.6) | 8.3 (1.8) | 8.9 (1.4) | 8.5 (2.2) | 5.6 (3.6) | 7.8 (2.2) | 4.1 (3.7) | 7.2 (2.9) | 7.2 (2.6) | 7.4 (2.3) | 8.9 (1.6) |
| Ireland | 10.0 (0) | 9.9 (0.2) | 10.0 (0) | 10.0 (0) | 10.0 (0) | 10.0 (0) | 10.0 (0) | 9.8 (0.5) | 9.7 (1.0) | 9.6 (1.0) | 10.0 (0) | 10.0 (0) | 9.9 (0.5) | 9.9 (0.2) | 9.4 (1.1) | 9.9 (0.5) | 5.9 (3.4) | 10.0 (0) | 9.7 (0.8) | 9.8 (0.6) | 9.9 (0.2) |
| Israel | 8.8 (1.2) | 9.0 (1.2) | 9.5 (0.9) | 9.7 (0.6) | 8.3 (1.8) | 9.5 (0.6) | 9.2 (0.8) | 9.1 (0.9) | 8.2 (1.6) | 8.3 (1.2) | 7.5 (1.6) | 9.2 (0.9) | 8.6 (1.1) | 9.5 (0.7) | 8.5 (1.7) | 8.4 (1.4) | 6.2 (2.4) | 8.2 (2.2) | 8.4 (1.4) | 8.5 (1.9) | 9.4 (0.6) |
| Italy | 8.9 (1.5) | 8.6 (2.1) | 8.7 (2.0) | 9.1 (1.5) | 8.4 (1.8) | 8.3 (1.7) | 9.0 (1.5) | 8.5 (1.6) | 8.3 (2.1) | 7.7 (2.0) | 7.8 (2.3) | 8.5 (2.0) | 8.3 (2.4) | 8.1 (2.3) | 6.6 (3.1) | 8.2 (1.9) | 4.9 (3.4) | 7.8 (2.6) | 8.1 (2.2) | 7.4 (2.5) | 8.4 (1.9) |
| Lebanon | 8.0 (1.3) | 9.1 (1.1) | 8.9 (0.7) | 8.6 (0.5) | 9.4 (0.5) | 9.4 (0.5) | 9.3 (0.8) | 9.0 (1.0) | 7.4 (1.0) | 7.0 (1.6) | 8.7 (1.0) | 9.0 (1.0) | 8.4 (0.8) | 9.4 (0.8) | 3.6 (4.3) | 7.7 (1.2) | 4.0 (3.9) | 7.3 (1.4) | 6.6 (1.4) | 7.7 (2.3) | 9.1 (0.7) |
| Latvia | 7.3 (3.1) | 7.3 (3.1) | 7.3 (2.5) | 7.7 (2.1) | 7.3 (1.5) | 7.3 (1.5) | 7.3 (1.5) | 7.3 (1.5) | 7.3 (1.5) | 7.3 (1.2) | 7.0 (1.0) | 7.3 (1.5) | 8.3 (1.5) | 3.0 (3.6) | 3.0 (3.6) | 8.0 (1.0) | 3.0 (3.6) | 8.0 (1.0) | 8.3 (1.2) | 8.7 (1.5) | 8.7 (1.5) |
| Lithuania | 9.6 (0.8) | 8.7 (1.8) | 9.0 (1.2) | 9.4 (1.0) | 9.4 (0.8) | 9.3 (1.1) | 8.9 (1.4) | 8.9 (1.4) | 7.7 (1.6) | 7.1 (2.0) | 9.0 (1.3) | 8.6 (1.4) | 9.4 (0.8) | 8.4 (1.6) | 0.3 (0.8) | 8.1 (1.6) | 7.3 (1.6) | 7.9 (1.8) | 7.9 (1.4) | 7.0 (1.9) | 8.7 (1.1) |
| Macedonia | 9.8 (0.5) | 10.0 (0) | 9.8 (0.5) | 9.8 (0.5) | 10.0 (0) | 10.0 (0) | 9.6 (0.9) | 9.8 (0.5) | 9.2 (1.3) | 8.5 (1.3) | 10.0 (0) | 9.8 (0.5) | 9.8 (0.5) | 9.8 (0.5) | 9.0 (1.7) | 9.4 (0.9) | 9.0 (1.2) | 9.6 (0.6) | 9.4 (0.9) | 9.6 (0.6) | 9.8 (0.5) |
| Malta | 8.0 (2.8) | 8.5 (2.1) | 9.5 (0.7) | 10.0 (0) | 10.0 (0) | 10.0 (0) | 8.5 (0.7) | 7.0 (1.4) | 6.5 (0.7) | 6.0 (0) | 10.0 (0) | 9.5 (0.7) | 8.0 (2.8) | 9.5 (0.7) | 0.5 (0.7) | 10.0 (0) | 0.0 (0) | 10.0 (0) | 8.0 (0) | 9.0 (1.4) | 10.0 (0) |
| Moldova | 9.5 (0.7) | 9.5 (0.7) | 9.0 (1.4) | 9.0 (1.4) | 9.5 (0.7) | 8.5 (2.1) | 9.0 (1.4) | 9.0 (1.4) | 7.5 (0.7) | 8.5 (2.1) | 7.5 (2.1) | 7.0 (0) | 8.0 (0) | 7.0 (0) | 7.0 (0) | 8.0 (0) | 7.0 (0) | 7.0 (0) | 8.0 (0) | 9.0 (0) | 9.0 (0) |
| Netherlands | 9.5 (1.0) | 9.4 (1.0) | 9.6 (1.0) | 9.5 (0.9) | 9.4 (1.0) | 9.7 (0.7) | 9.7 (0.8) | 9.4 (0.9) | 8.3 (1.2) | 7.9 (1.3) | 8.6 (1.3) | 9.5 (0.9) | 9.5 (0.9) | 9.5 (1.8) | 9.5 (0.9) | 9.3 (0.9) | 5.6 (3.8) | 9.2 (0.9) | 9.2 (1.1) | 9.4 (0.9) | 9.5 (0.8) |
| Norway | 9.4 (0.9) | 9.4 (1.0) | 9.5 (0.9) | 9.6 (0.7) | 9.2 (1.3) | 9.3 (1.1) | 9.5 (1.0) | 9.4 (1.1) | 8.2 (1.7) | 7.9 (1.7) | 8.9 (1.2) | 9.6 (0.7) | 9.1 (1.1) | 9.9 (0.5) | 9.2 (1.2) | 7.8 (2.0) | 8.8 (1.3) | 9.2 (1.4) | 8.5 (1.4) | 8.7 (1.4) | 9.5 (0.7) |
| Poland | 8.3 (1.8) | 8.5 (1.9) | 8.2 (2.1) | 9.2 (1.5) | 8.2 (2.0) | 8.4 (1.6) | 8.4 (1.7) | 8.5 (1.6) | 7.9 (2.0) | 7.0 (2.3) | 8.1 (1.9) | 7.1 (2.7) | 8.2 (2.1) | 6.5 (3.3) | 2.3 (3.1) | 7.4 (2.3) | 3.3 (3.3) | 6.5 (2.8) | 7.1 (2.5) | 6.6 (3.0) | 8.5 (1.9) |
| Portugal | 9.2 (1.7) | 9.1 (1.7) | 9.4 (0.9) | 9.6 (0.7) | 9.5 (1.0) | 9.6 (1.0) | 9.5 (0.7) | 9.5 (0.9) | 9.0 (1.2) | 8.0 (1.9) | 9.3 (1.2) | 9.3 (1.0) | 9.4 (1.2) | 9.6 (0.9) | 6.8 (3.1) | 9.2 (1.1) | 6.6 (2.8) | 8.5 (1.9) | 7.9 (1.8) | 8.8 (1.2) | 9.1 (0.9) |
| Romania | 9.5 (0.9) | 8.7 (2.0) | 8.8 (1.5) | 9.6 (1.0) | 9.3 (1.4) | 9.4 (0.8) | 8.9 (1.3) | 9.2 (1.0) | 8.1 (1.8) | 7.5 (2.2) | 9.3 (1.1) | 9.0 (1.5) | 9.0 (1.5) | 7.4 (2.8) | 3.8 (3.8) | 8.2 (2.0) | 5.1 (3.2) | 7.6 (2.7) | 7.7 (2.2) | 8.1 (2.2) | 9.0 (1.7) |
| Russia | 8.9 (1.7) | 8.6 (1.6) | 8.7 (1.5) | 9.4 (1.0) | 9.2 (1.2) | 9.0 (1.5) | 8.6 (1.8) | 8.6 (2.0) | 8.1 (2.4) | 7.5 (2.5) | 8.6 (1.6) | 7.3 (2.9) | 9.1 (1.2) | 8.2 (2.4) | 3.8 (3.6) | 8.2 (1.5) | 4.5 (3.6) | 8.3 (2.0) | 8.4 (2.0) | 8.4 (2.1) | 9.2 (1.5) |
| Serbia | 9.6 (1.6) | 9.0 (2.2) | 8.9 (2.4) | 9.0 (2.4) | 8.9 (2.7) | 8.8 (2.5) | 8.7 (2.3) | 8.9 (2.3) | 8.1 (2.5) | 7.6 (2.8) | 8.6 (2.7) | 8.5 (2.3) | 8.9 (2.0) | 8.4 (2.9) | 4.6 (3.8) | 8.4 (2.1) | 4.2 (4.3) | 8.6 (2.0) | 8.7 (1.9) | 8.4 (1.8) | 9.2 (1.6) |
| Slovakia | 9.5 (1.0) | 9.5 (0.7) | 9.5 (0.8) | 9.4 (1.3) | 9.3 (1.0) | 9.3 (0.9) | 9.4 (1.0) | 9.4 (1.0) | 8.9 (1.3) | 8.7 (1.6) | 9.3 (0.9) | 7.8 (2.5) | 9.4 (1.0) | 9.3 (1.2) | 7.0 (3.1) | 8.3 (1.5) | 7.3 (2.6) | 8.6 (1.5) | 8.3 (1.7) | 8.3 (1.8) | 8.6 (1.7) |
| Slovenia | 9.6 (0.7) | 9.6 (0.5) | 9.9 (0.3) | 9.8 (0.4) | 8.3 (3.6) | 8.6 (3.0) | 9.8 (0.4) | 9.6 (0.5) | 8.4 (1.6) | 7.8 (1.8) | 9.0 (1.3) | 9.7 (0.7) | 9.4 (1.2) | 9.7 (0.7) | 5.9 (3.8) | 5.8 (2.7) | 5.6 (3.4) | 7.9 (2.6) | 8.1 (2.2) | 8.4 (1.8) | 9.8 (0.4) |
| Spain | 9.0 (1.3) | 9.1 (1.2) | 9.3 (1.0) | 9.4 (0.9) | 9.0 (1.3) | 9.5 (0.8) | 9.2 (1.1) | 9.1 (1.3) | 8.1 (2.1) | 7.6 (2.2) | 8.8 (1.6) | 8.9 (2.0) | 8.8 (1.6) | 9.6 (1.0) | 8.7 (1.9) | 8.9 (1.2) | 6.8 (2.6) | 8.4 (1.8) | 7.6 (2.0) | 8.1 (1.8) | 8.8 (1.2) |
| Sweden | 9.0 (1.1) | 9.3 (0.9) | 9.5 (0.9) | 9.7 (0.6) | 8.2 (2.1) | 9.0 (1.6) | 9.5 (0.7) | 9.1 (1.2) | 7.8 (1.9) | 7.4 (2.3) | 8.2 (1.7) | 9.3 (1.4) | 9.1 (1.1) | 9.8 (0.9) | 3.6 (3.4) | 5.7 (2.4) | 4.5 (3.4) | 8.5 (2.0) | 8.3 (1.8) | 8.3 (2.5) | 9.5 (0.9) |
| Switzerland | 9.6 (0.8) | 9.4 (1.0) | 9.7 (0.5) | 9.9 (0.3) | 9.7 (0.6) | 9.7 (0.8) | 9.7 (0.8) | 9.7 (0.7) | 8.4 (1.3) | 7.7 (1.2) | 9.4 (0.9) | 9.7 (0.6) | 9.4 (0.8) | 9.8 (1.0) | 8.3 (2.9) | 9.3 (0.9) | 7.8 (3.0) | 9.5 (0.7) | 8.3 (1.4) | 8.8 (1.1) | 9.5 (0.9) |
| Turkey (through internal medicine) | 8.8 (1.2) | 8.7 (1.2) | 8.7 (1.3) | 9.1 (1.2) | 7.8 (1.9) | 8.8 (1.5) | 8.9 (1.3) | 9.1 (1.1) | 8.5 (1.4) | 7.9 (1.9) | 7.9 (2.3) | 8.9 (1.6) | 8.0 (2.2) | 8.8 (2.0) | 4.3 (3.6) | 7.9 (1.5) | 5.7 (3.0) | 7.0 (2.9) | 7.1 (2.3) | 7.3 (2.2) | 8.2 (1.9) |
| Turkey (through physical therapy) | 9.8 (0.4) | 9.8 (0.4) | 9.5 (0.5) | 9.8 (0.5) | 9.7 (0.7) | 9.3 (0.8) | 9.4 (0.5) | 9.8 (0.4) | 8.8 (0.7) | 7.6 (1.3) | 9.5 (1.0) | 9.8 (0.5) | 9.5 (0.8) | 9.8 (0.4) | 6.6 (3.3) | 9.3 (0.7) | 7.3 (2.4) | 8.3 (1.5) | 8.8 (0.9) | 8.7 (1.2) | 9.2 (1.1) |
| United Kingdom | 9.7 (0.8) | 9.7 (0.8) | 9.9 (0.3) | 9.9 (0.4) | 9.9 (0.4) | 9.8 (0.5) | 9.9 (0.4) | 9.7 (0.6) | 9.3 (1.1) | 9.0 (1.3) | 9.2 (1.1) | 9.8 (0.5) | 9.7 (0.6) | 10.0 (0.2) | 6.5 (3.7) | 9.2 (1.4) | 4.2 (3.2) | 9.5 (1.1) | 8.1 (2.2) | 9.2 (1.1) | 9.8 (0.6) |
| Ukraine | 7.3 (2.5) | 7.7 (2.5) | 8.0 (1.7) | 8.7 (1.5) | 8.7 (1.5) | 8.0 (1.7) | 8.3 (2.1) | 7.7 (2.5) | 8.0 (2.8) | 7.3 (2.3) | 9.0 (1.0) | 8.0 (1.0) | 7.7 (2.1) | 3.7 (4.7) | 4.3 (4.0) | 7.3 (2.1) | 4.7 (4.0) | 7.7 (2.3) | 8.0 (2.7) | 9.0 (1.0) | 9.0 (1.0) |

Mean (SD)

* These competences refer to the management of a patient with the given disease or treatment
