# Supplementary material for: Rheumatology training experience across Europe: analysis of core competences
Source: Arthritis Res Ther. 2016 Sep 23;18:213. doi: 10.1186/s13075-016-1114-y (PMC5035447; doi:10.1186/s13075-016-1114-y)
Supplement: Additional file 9: Table S8. — Factors associated with self-reported ability and with limited experience in early rheumatoid arthritis/undifferentiated arthritis (≤10 patients) and in performing knee aspiration (≤10 procedures). (DOCX 18 kb) [file 13075_2016_1114_MOESM9_ESM.docx]

Additional file 9

Table 4: Model for self-reported ability in early rheumatoid arthritis/undifferentiated arthritis and in performing a knee aspiration

|  |  | Self-reported ability in managing a patient with early RA/undifferentiated arthritis  Regression coefficient  (95% confidence interval) | Self-reported ability in performing a knee aspiration  Regression coefficient  (95% confidence interval) |
| --- | --- | --- | --- |
| Age | ≤ 25 years | Reference | Reference |
|  | 26-30 years | 0.73 (0.06, 1.39) | -0.34 (-1.21, 0.53) |
|  | 31-35 years | 0.72 (0.03, 1.40) | -0.46 (-1.37, 0.44) |
|  | 36-40 years | 0.79 (0.07, 1.51) | -0.41 (-1.35, 0.53) |
|  | >40 years | 0.71 (-0.05, 1.46) | -0.24 (-1.22, 0.74) |
| Male vs female | | -0.20 (-0.37, -0.03) | -0.08 (-0.29, 0.13) |
| Trainee vs rheumatologist | | 0.25 (0.06, 0.44) | 0.13 (-0.11, 0.36) |
| Country* | UK | Reference | Reference |
|  | Albania | -2.62 (-3.55, -1.69) | -1.90 (-3.15, -0.66) |
|  | Armenia | -1.05 (-3.45, 1.34) | 1.70 (-1.24, 4.65) |
|  | Austria | -0.12 (-0.81, 0.57) | -0.22 (-1.09, 0.65) |
|  | Belarus | 0.15 (-1.27, 1.56) | -1.25 (-3.01, 0.51) |
|  | Belgium | -0.55 (-1.26, 0.16) | 0.26 (-0.61, 1.12) |
|  | Bosnia | 0.42 (-0.56, 1.41) | -0.39 (-1.62, 0.83) |
|  | Bulgaria | -0.14 (-1.06, 0.78) | -0.64 (-1.77, 0.49) |
|  | Croatia | -0.49 (-1.46, 0.48) | -0.88 (-2.08, 0.32) |
|  | Czech Rep | -0.92 (-1.49, -0.36) | -0.54 (-1.22, 0.15) |
|  | Denmark | -0.08 (-0.54, 0.38) | -0.08 (-0.63, 0.47) |
|  | Estonia | -0.21 (-1.15, 0.72) | -0.44 (-1.59, 0.71) |
|  | Finland | -0.49 (-1.26, 0.28) | -0.21 (-1.13, 0.71) |
|  | France | -1.44 (-1.91, -0.97) | -0.44 (-1.01, 0.13) |
|  | Georgia | -0.55 (-1.62, 0.52) | -1.99 (-3.32, -0.66) |
|  | Germany | -0.20 (-0.88, 0.48) | -0.59 (-1.43, 0.24) |
|  | Greece | -0.87 (-1.40, -0.33) | -0.44 (-1.10, 0.21) |
|  | Hungary | -0.86 (-1.41, -0.32) | -0.83 (-1.51, -0.16) |
|  | Ireland | 0.11 (-0.58, 0.80) | -0.04 (-0.88, 0.79) |
|  | Israel | -0.43 (-1.11, 0.24) | -0.57 (-1.43, 0.29) |
|  | Italy | -0.86 (-1.58, -0.14) | -1.61 (-2.49, -0.73) |
|  | Lebanon | -0.06 (-1.03, 0.92) | 0.55 (-0.65, 1.75) |
|  | Latvia | -2.55 (-3.96, -1.14) | -4.27 (-6.03, -2.51) |
|  | Lithuania | -0.69 (-1.75, 0.36) | 0.80 (-0.45, 2.05) |
|  | Macedonia | -0.19 (-1.31, 0.92) | -0.31 (-2.04, 1.41) |
|  | Malta | -0.90 (-2.61, 0.81) | -0.42 (-2.52, 1.67) |
|  | Moldova | -1.75 (-4.13, 0.63) | -0.54 (-3.48, 2.40) |
|  | Netherlands | -0.03 (-0.63, 0.57) | -0.30 (-1.01, 0.42) |
|  | Norway | -0.20 (-0.73, 0.32) | -0.07 (-0.71, 0.58) |
|  | Poland | -1.24 (-1.74, -0.74) | -2.42 (-3.04, -1.80) |
|  | Portugal | -0.10 (-0.64, 0.44) | 0.00 (-0.66, 0.66) |
|  | Romania | -0.82 (-1.32, -0.31) | -0.62 (-1.27, 0.02) |
|  | Russia | -1.16 (-1.81, -0.50) | -1.05 (-1.85, -0.25) |
|  | Serbia | -0.97 (-1.71, -0.24) | -0.67 (-1.57, 0.23) |
|  | Slovakia | -0.41 (-1.11, 0.28) | -0.51 (-1.36, 0.35) |
|  | Slovenia | 0.04 (-0.79, 0.87) | -0.29 (-1.31, 0.73) |
|  | Spain | -0.51 (-0.99, -0.03) | -0.28 (-0.86, 0.30) |
|  | Sweden | -0.29 (-0.94, 0.36) | 0.11 (-0.67, 0.90) |
|  | Switzerland | -0.16 (-0.76, 0.44) | -0.20 (-0.93, 0.53) |
|  | Turkey (GIM) | -0.80 (-1.34, -0.27) | -1.00 (-1.65, -0.36) |
|  | Turkey (Physical therapy) | -0.40 (-1.17, 0.38) | -0.10 (-1.08, 0.88) |
|  | Ukraine | -1.54 (-2.95, -0.13) | -4.41 (-6.15, -2.67) |
| Education (yes vs no) | | 0.54 (0.28, 0.81) | 0.57 (0.32, 0.82) |
| Patient exposure (>10 vs ≤10) | | 0.88 (0.60, 1.15) | 2.51 (2.18, 2.83) |
| Assessment (yes vs no) | | -0.05 (-0.22,0.12) | 0.16 (-0.04, 0.37) |

* p<0.001 overall effect of the variable country (all dummies in the model). UK was consdered as the reference country

GIM: general internal medicine

Table: Factors associated with limited experience in early rheumatoid arthritis/undifferentiated arthritis (≤10 patients) and in performing a knee aspiration (≤10 procedures)

|  |  | Limited patient experience (≤10) in early rheumatoid arthritis/undifferentiated arthritis  Odd Ratio  (95% confidence interval) | Limited experience (≤10) in performing a knee aspiration  Odd Ratio  (95% confidence interval) |
| --- | --- | --- | --- |
| Age | ≤ 25 years | Reference | Reference |
|  | 26-30 years | 2.73 (0.90, 8.29) | 12.34 (2.60, 58.62) |
|  | 31-35 years | 4.76 (1.33, 17.05) | 22.71 (4.30, 120.00) |
|  | 36-40 years | 6.60 (1.58, 27.58) | 23.38 (4.13, 132.30) |
|  | >40 years | 2.30 (0.48, 11.03) | 9.00 (1.45, 55.85) |
| Male vs female | | 0.74 (0.44, 1.24) | 1.74 (1.03, 2.93) |
| Trainee vs rheumatologist | | 2.43 (1.30, 4.56) | 2.78 (1.59, 4.85) |
| Country* | UK | Reference | Reference |
|  | Albania | 0.10 (0.01, 1.42) | NA |
|  | Armenia | NA | 1.39 (0.03, 60.51) |
|  | Austria | 0.63 (0.04, 10.92) | 3.14 (0.21, 46.62) |
|  | Belarus | NA | NA |
|  | Belgium | 0.16 (0.01, 1.77) | 11.51 (0.68, 194.47) |
|  | Bosnia | 0.02 (0.00, 0.28) | NA |
|  | Bulgaria | 0.32 (0.02, 6.31) | 3.16 (0.17, 57.78) |
|  | Croatia | 0.28 (0.01, 5.33) | 1.70 (0.09, 33.74) |
|  | Czech Rep | 0.10 (0.01, 0.88) | 8.70 (0.60, 125.88) |
|  | Denmark | 0.74 (0.08, 7.14) | 107.49 (5.98, 1933.25) |
|  | Estonia | NA | 5.55 (0.27, 113.67) |
|  | Finland | NA | NA |
|  | France | 0.29 (0.03, 2.38) | 130.25 (8.29, 2046.73) |
|  | Georgia | 0.05 (0.00, 0.75) | 0.36 (0.01, 10.00) |
|  | Germany | NA | 16.17 (0.86, 302.34) |
|  | Greece | 0.36 (0.04, 3.73) | 10.73 (0.75, 154.39) |
|  | Hungary | 0.06 (0.01, 0.53) | 3.42 (0.25, 46.07) |
|  | Ireland | NA | NA |
|  | Israel | 0.36 (0.03, 4.56) | NA |
|  | Italy | NA | 14.92 (0.79, 282.53) |
|  | Lebanon | 0.14 (0.01, 1.98) | 4.57 (0.24, 88.56) |
|  | Latvia | NA | NA |
|  | Lithuania | NA | NA |
|  | Macedonia | NA | NA |
|  | Malta | NA | NA |
|  | Moldova | NA | 1.83 (0.04, 84.01) |
|  | Netherlands | 0.34 (0.03, 4.07) | 52.95 (2.11, 1330.69) |
|  | Norway | 1.31 (0.08, 22.54) | NA |
|  | Poland | 0.25 (0.03, 2.31) | 2.89 (0.22, 37.65) |
|  | Portugal | 0.31 (0.03, 2.91) | 20.47 (1.39, 302.25) |
|  | Romania | 0.44 (0.05, 4.15) | 1.18 (0.09, 15.50) |
|  | Russia | 0.06 (0.01, 0.56) | 2.20 (0.16, 30.91) |
|  | Serbia | NA | 5.56 (0.35, 88.13) |
|  | Slovakia | NA | 15.14 (0.80, 286.17) |
|  | Slovenia | NA | NA |
|  | Spain | 1.13 (0.10, 13.32) | 160.34 (6.47, 3974.92) |
|  | Sweden | NA | NA |
|  | Switzerland | 0.32 (0.03, 3.82) | 45.90 (1.83, 1150.06) |
|  | Turkey (GIM) | NA | 18.97 (1.18, 305.22) |
|  | Turkey (Physical therapy) | NA | NA |
|  | Ukraine | NA | NA |

*p<0.0001 for overall effect of country variable (with all dummies) in the models

GIM: general internal medicine; NA: not available, a regression coefficient could not be estimated for these countries, because in all of them there was a very low proportion of limited patients experience in these competences (most of them even 0 cases); this means that the country of training had a perfect association with the outcome in these cases (one can derive the outcome from the independent variable), therefore being impossible to compute a regression coefficient.
